# 748 Accidental Thermal Injury From Ignition of Home Oxygen: Is Burn Center Admission Advantageous?

**DOI:** 10.1093/jbcr/irad045.223

**Published:** 2023-05-15

**Authors:** John Bailey, Hope Werenski, Anju Saraswat, James Holmes, Rachel McEathron, Christopher Craig, Jeff Williams

**Affiliations:** Wake Forest School of Medicine, Winston-Salem, North Carolina; Wake Forest University, School of Medicine, Winston-Salem, North Carolina; Atrium Wake Forest Baptist Medical Center, Winston-Salem, North Carolina; Wake Forest School of Medicine, Winston-Salem, North Carolina; Wake Forest University, School of Medicine, Winston-Salem, North Carolina; Atrium Wake Forest Baptist Medical Center, Winston-Salem, North Carolina; Wake Forest School of Medicine, Winston-Salem, North Carolina; Wake Forest University, School of Medicine, Winston-Salem, North Carolina; Atrium Wake Forest Baptist Medical Center, Winston-Salem, North Carolina; Wake Forest School of Medicine, Winston-Salem, North Carolina; Wake Forest University, School of Medicine, Winston-Salem, North Carolina; Atrium Wake Forest Baptist Medical Center, Winston-Salem, North Carolina; Wake Forest School of Medicine, Winston-Salem, North Carolina; Wake Forest University, School of Medicine, Winston-Salem, North Carolina; Atrium Wake Forest Baptist Medical Center, Winston-Salem, North Carolina; Wake Forest School of Medicine, Winston-Salem, North Carolina; Wake Forest University, School of Medicine, Winston-Salem, North Carolina; Atrium Wake Forest Baptist Medical Center, Winston-Salem, North Carolina; Wake Forest School of Medicine, Winston-Salem, North Carolina; Wake Forest University, School of Medicine, Winston-Salem, North Carolina; Atrium Wake Forest Baptist Medical Center, Winston-Salem, North Carolina

## Abstract

**Introduction:**

Home oxygen therapy is available for the supportive treatment of patients with chronic lung disease. Nevertheless, complications associated with home oxygen use have been well described. The typical injury pattern associated with the accidental ignition of home oxygen does not mandate emergent intubation. However, due to the thermal component of the injury, patients are initially directed towards admission to burn units. The recent stress applied to the healthcare system by COVID-19 has highlighted the potential benefit of flexibility in the disposition of injured patients. In particular, we wanted to determine if there is a demonstrable benefit from admission to an American Burn Association (ABA) verified burn center for patients presenting with home oxygen ignition injuries.

**Methods:**

After obtaining approval from our local Institutional Review Board, we conducted a retrospective review of our institution's ABA-verified burn center database from January 2016 through May 2022. Individual charts were inspected for discrete data points related to the general descriptor of the patient's injuries, comorbidities, and hospital course. Using independent samples t-tests, we compared patients admitted to the burn service with those primarily cared for by non-burn service teams.

**Results:**

We identified 49 adult patients admitted with burn injuries associated with home oxygen use during the study period. These patients were divided into those intubated at or near the time of admission and those managed without intubation. Of the 29 patients intubated on admission, the burn service managed 19 of these patients, and non-burn services managed 10. There were no identified differences in the outcomes of ventilator days, days in the ICU, total length of stay, or mortality between the two groups. Similarly, we examined the 20 patients admitted without intubation. The burn service managed 7 of these patients, and non-burn services managed 13. Again, the two groups had no identified differences in the outcomes of days in the ICU, total length of stay, or mortality.

**Conclusions:**

This single-center review revealed no difference in outcomes between patients cared for in an ABA-verified burn center versus non-burn services for home oxygen ignition injury. From a care perspective and resource utilization, the outcomes appear similar. Further, we note that patients required only brief periods of mechanical ventilation – supportive of the opportunity to avoid intubation in these patients. Prevention efforts, like those in the burn center, need to continue, and further studies must be done to confirm these preliminary findings.

**Applicability of Research to Practice:**

These findings are supportive of potential opportunities to wisely use Burn Center capacity.

